# Prelimbic cortex responds to male ultrasonic vocalizations in the presence of a male pheromone in female mice

**DOI:** 10.3389/fncir.2022.956201

**Published:** 2022-09-28

**Authors:** Akari Asaba, Kensaku Nomoto, Takuya Osakada, Tomohiko Matsuo, Ko Kobayakawa, Reiko Kobayakawa, Kazushige Touhara, Kazutaka Mogi, Takefumi Kikusui

**Affiliations:** ^1^Department of Animal Science and Biotechnology, School of Veterinary Medicine, Azabu University, Sagamihara, Japan; ^2^Department of Physiology, Dokkyo Medical University School of Medicine, Mibu, Tochigi, Japan; ^3^Department of Applied Biological Chemistry, Graduate School of Agricultural and Life Sciences, The University of Tokyo, Bunkyo, Tokyo, Japan; ^4^Institute of Biomedical Science, Kansai Medical University, Hirakata, Osaka, Japan

**Keywords:** ultrasonic vocalizations, male pheromone, immediate early gene, medial prefrontal cortex, prelimbic cortex, mice, mouse behavior

## Abstract

Sensory signals are critical to perform adaptive social behavior. During copulation, male mice emit ultrasonic vocalizations (USVs). Our previous studies have shown that female mice exhibit approach behavior toward sound sources of male USVs and that, after being exposed to a male pheromone, exocrine gland-secreting peptide 1 (ESP1), female mice exhibited a preference toward a particular type of male USVs. These findings suggest that male USVs modulate female courtship behavior. However, it remains unclear which brain regions and what cell types of neurons are involved in neuronal processing of male USVs. To clarify this issue, immediate early gene analysis, behavioral analysis, and neurochemical analysis were performed. The *in situ* hybridization analysis of *c-fos* mRNA in multiple brain regions showed that neurons in the prelimbic cortex were responsive to presentation of male USVs in the presence of ESP1. Furthermore, this study found that activity of prelimbic cortex was correlated with the duration of female exploration behavior toward a sound source of the USVs. Finally, by using double immunohistochemistry, the present study showed that the prelimbic neurons responding to the presentation of male USVs were presumably excitatory glutamatergic neurons. These results suggest that the prelimbic cortex may facilitate female courtship behavior in response to male USVs.

## Introduction

Sensory signals are important for performing adaptive social behavior. For example, previous studies showed that mother rodents approached a sound source of pup ultrasonic vocalizations (USVs) more in the presence of pup odor (Farrell and Alberts, 2002; Okabe et al., 2013). In addition, electrophysiological studies reported that neural responses to pup USVs in the auditory cortex of lactating mothers increased when they were presented with pup odor (Cohen et al., 2011). These results indicate that rodents use various sensory information for social behavior.

Male mice emit USVs when they encounter female mice (Whitney et al., 1973; Nyby et al., 1977). Studies showed that male USVs induced approach behavior of female mice (Pomerantz et al., 1983; Hammerschmidt et al., 2009; Asaba et al., 2017; Nomoto et al., 2018). In addition, our previous study showed that male USVs activated female kisspeptin neurons in the hypothalamus that regulate the secretion of gonadotropin releasing hormone (Asaba et al., 2017), thus enhancing the reproductive function. Consequently, male USVs are thought to serve as courtship songs.

Besides approach behavior, female mice exhibit a preference toward a particular type of male USVs. Studies showed that female mice stayed longer near a sound source of one type of USVs than that of another type of USVs in experiments in which two USVs were played back simultaneously (Musolf et al., 2010; Asaba et al., 2014; Nomoto et al., 2020). Specifically, our previous studies demonstrated that female mice preferred male USVs of a different strain than themselves (Asaba et al., 2014; Nomoto et al., 2020). These studies also revealed that pre-exposure of a male pheromone, exocrine gland-secreting peptide 1 (ESP1) (Haga et al., 2010), was necessary for females’ USVs preferences.

These findings indicate that male USVs modulate female courtship behavior and that female mice are able to acquire complicated information such as the strain identity of senders of USVs. However, it remains to be clarified which brain regions and what types of neurons are involved in the sensory processing of male USVs in the female brain. By combining immediate early gene analysis, behavioral analysis, and neurochemical analysis, this study showed that neurons in the prelimbic cortex that were presumably excitatory were activated by male USVs in the presence of ESP1 in female mice.

## Materials and methods

### Animals

Female mice of the C57BL/6J strain (B6) were used (purchased from CLEA Japan, Inc., Tokyo, Japan, and maintained in the laboratory). Data from 48 B6 mice was used for the analysis (12–30 weeks old; *N* = 12, 35, 1 for Experiment 1, 2, 3, respectively). All animals were housed in groups (cage size, 175 mm × 245 mm × 125 mm). The light schedule was a regular 12-h light/dark cycle (lights on at 06:00). All procedures were performed in accordance with the guidelines of “Policies Governing the Use of Live Vertebrate Animals” of Azabu University, and were approved by The Ethical Committee for Vertebrate Experiments of Azabu University (ID #090219).

### Preparation of ultrasonic vocalizations for playback

The sound source recorded in the previous study was used (Asaba et al., 2015; Nomoto et al., 2020). One male BALB/c mouse was used for recording USVs (purchased from CLEA Japan, Inc., Tokyo, Japan). To promote vocalizations, in a sound-attenuated chamber, the male mouse was paired with a female C57BL/6 mouse, which was ovariectomized and devocalized. Sexual receptivity of the female mouse was hormonally induced. A microphone (CM16/CMPA, Avisoft Bioacoustics, Glienicke/Nordbahn, Germany) capable of recording USVs was placed through the wire mesh of the cage. The USVs were digitally converted, filtered using a 20–145.8 kHz band-pass filter, and recorded at a sampling rate of 300 kHz (UltraSoundGate 116H and RECORDER USGH, Avisoft Bioacoustics, Glienicke/Nordbahn, Germany). A fragment of 20 s was extracted from the original sound file (Audition 3.0, Adobe, CA, USA). Ambient noises were digitally reduced. A silent part of the sound file was used as background noise.

### Playback experiments

The behavioral experiments were performed in a plastic cage (33.8 cm × 22.5 cm × 14.0 cm). Speakers [nc-Si emitters (Uematsu et al., 2007); Kato Acoustics Consulting Office, Yokohama, Japan] were placed behind copper meshes (hole diameter, 6 cm) that were located on smaller walls of the cage. Female mice were reared in the behavioral apparatus for at least 7 days and were acclimatized to the apparatus sufficiently. Because our previous studies had shown that non-estrous females exhibited USVs preference behavior (Asaba et al., 2014), only non-estrous females for the playback experiments were used. Vaginal lavage was collected daily between 11:00 and 14:00 to check the estrous status of female mice (McLean et al., 2012).

On the day of the experiment, the behavioral cage containing a female mouse was moved to a soundproof box, except for food and water. The animals were acclimatized to the experimental situation for 6 h (Experiment 1) or 4 h (Experiment 2 and 3). Thirty minutes before the playback of male USVs, female mice were exposed to a piece of cotton containing recombinant ESP1 (20 μg) in 0.1 M Tris buffer solution. In Experiment 2, a piece of cotton containing 0.1 M Tris buffer solution was used as a control stimulus. Then, male USVs or background noise were presented for 6 min (Experiment 1) or 20 min (4 min 30 s playback, 30 s stop, repeated four times) (Experiments 2 and 3).

#### *In situ* hybridization

*In situ* hybridization followed a protocol in a previous study (Matsuo et al., 2015). Thirty minutes after the sound presentation, the female mice were deeply anesthetized with isoflurane and perfused transcardially with 0.1 M phosphate-buffered saline (PBS) followed by 4% paraformaldehyde (PFA) in 0.1 M PBS. The brains were extracted and immersed in 4% PFA in 0.1 M PBS overnight at 4°C. Then, the brains were dehydrated in a series of graded ethanol and xylene solution and embedded in paraffin using an automated system (Sakura rotary, RH-12DM, Sakura Finetek, Tokyo, Japan). Coronal slices with a thickness of 5 μm were prepared using an automatic slide preparation system (AS-200S, Kurabo, Osaka, Japan). *In situ* hybridization was performed using an automated system (Discovery XT, Ventana Medical Systems, Basel, Switzerland). Brain slices were stained and analyzed at a 90-μm interval. To prepare antisense RNA probes for the *c-fos* gene, DNA fragments spanning the –129- to 537-bp and the 543- to 1,152-bp regions of the *c-fos* gene were amplified by PCR from brain cDNA from C57BL/6 mice. The digoxigenin (DIG)-labeled probe for the *c-fos* gene (1:200 dilution) was hybridized for 3 h using a RiboMap Kit (Roche, Basel, Switzerland) at 74°C. The slides were then incubated with a biotin-conjugated anti-DIG antibody (1:500; Jackson ImmunoResearch, PA, USA) at 37°C for 28 min. The probe was detected using the Ventana BlueMap Kit (Roche, Basel, Switzerland) at 37°C for 6 h and counterstained with a Red counterstain kit (Roche, Basel, Switzerland) at 37°C for 4 min. Coverslips were applied using an automated system (Tissue-Tek Glas; Sakura Finetek, Tokyo, Japan). The stained images were scanned using a NanoZoomer virtual microscope system (2.0 RS, Hamamatsu Photonics, Hamamatsu, Japan). For quantification analysis, images were converted to grayscale, and signal intensities were quantified as the *c-fos* mRNA expression level. A 0.25 mm × 0.25 mm area was selected from each brain region, and the average of the left and right sides was calculated.

### Immunohistochemistry

Immunohistochemical protocols followed a previous study (Asaba et al., 2017). Ninety minutes after the presentation of the sound, female mice were deeply anesthetized with isoflurane and perfused transcardially with 0.1 M PBS followed by 4% PFA in 0.1 M PBS. The brains were extracted from the skull, postfixed overnight with 4% PFA in 0.1 M PBS, and then immersed in 30% sucrose solution in 0.1 M PBS until they sank for cryopreservation. Coronal slices with a thickness of 30 μm were prepared using a cryostat (CM1850, Wetzlar, Germany). Sections containing the medial prefrontal cortex were immunostained. Brain slices were first incubated with 5% normal donkey serum (Jackson Immunoresearch, PA, USA) in 0.1 M PBS solution containing 0.3% Triton X-100 (PBST) for 60 min. Then, the slices were incubated with anti-c-fos antibody (1:250; SC52-G, Santa Cruz Biotechnology, CA, USA) in the blocking solution at 4°C for 48 h. Then, the slices were incubated with anti-goat IgG antibody conjugated to Alexa-594 (1:250; Jackson ImmunoResearch, PA, USA) at 4°C for 24 h. Slices were washed with PBST between steps. Stained slices were attached to glass slides with a mounting medium containing nuclear staining (DAPI-Fluoromount-G, Southern Biotech, AL, USA). In Experiment 3, in addition to anti-c-fos antibody, anti-GABA antibody (1:5,000; A2052, Sigma-Aldrich, MO, USA) or anti-GLS2 (phosphate activated glutaminase 2) antibody (1:1,000, ab113509, Abcam, Cambridge, United Kingdom) was added to the primary antibody solution. GABA and GLS2 (Kaneko and Mizuno, 1994) were used as markers for inhibitory GABAergic and excitatory glutamatergic neurons, respectively. Anti-rabbit IgG antibody conjugated to Alexa-488 (1:250; Jackson ImmunoResearch, PA, USA) was added to the secondary antibody solution. The stained images were photographed using a fluorescence microscope (BX51-N, Olympus, Tokyo, Japan). The number of immunoreactive neurons were counted manually by a person blind to experimental conditions.

### Behavioral analysis

Female behavior was recorded with a camera installed in the soundproof box for 30 min (from 5 min before the start of playback to 5 min after the end of playback). The 30-min video was divided into six time windows of 5 min each, and then the following behaviors were analyzed using a custom-made Excel macro.

(1) Initial latency and duration of speaker exploration behavior (wire mesh in front of the speaker).
(2) Time spent in the speaker zone (the half side where the speaker is placed was defined as the speaker zone).
(3) Duration of immobility and sleep.

### Statistical analysis

SPSS (IBM, NY, USA) and JMP 12 (SAS institute, NC, USA) were used for data analysis and statistical analysis. Statistical analysis was carried out using Welch’s *t*-test, two-way ANOVA followed by *post hoc* Bonferroni test, and Tukey HSD test. The significance level was set at 0.05. In Experiment 1, since there were 79 comparisons, the significance levels were adjusted by controlling false discovery rate <0.05 (Benjamini et al., 2001).

## Results

### Experiment 1

After being exposed to the male pheromone, ESP1, female mice were presented with male USVs (ESP1+USVs group; *N* = 6) or noise (ESP1+noise group; *N* = 6). Expression levels of *c-fos* mRNA were analyzed in various brain regions.

#### Olfactory cortex

**FIGURE 1:**
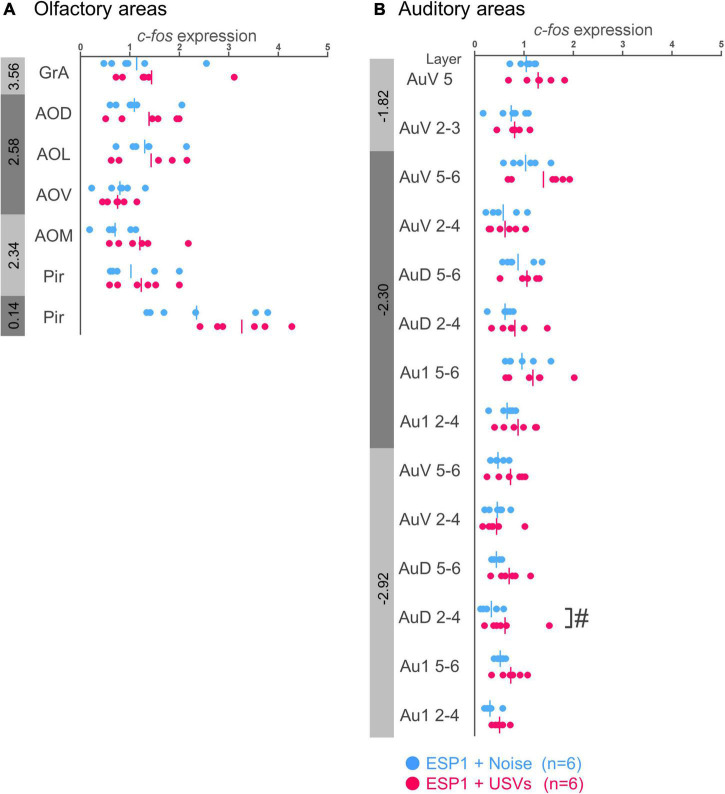
Expression levels of *c-fos* mRNA in the olfactory **(A)** and auditory **(B)** cortices of female mice. Blue and red symbols represent individual data for ESP1+noise and (exocrine gland-secreting peptide 1) ESP1+USVs (ultrasonic vocalizations) conditions, respectively. Vertical lines indicate mean values. Numbers in gray bands indicate approximate anteroposterior distance to the bregma (mm). ^#^*p* < 0.05 (Welch’s *t*-test; but not significant after multiple comparisons correction). GrA, granule cell layer of the accessory olfactory bulb; AOD, anterior olfactory area, dorsal part; AOL, anterior olfactory area, lateral part; AOV, anterior olfactory area, ventral part; AOM, anterior olfactory area, medial part; Pir, piriform cortex; AuV, secondary auditory cortex, ventral area; AuD, secondary auditory cortex, dorsal area; Au1, primary auditory cortex.

There were no significant differences between the ESP1+USVs and ESP1+noise groups in any subregion (Figure 1A).

#### Auditory cortex

In the layers 2–4 of the secondary auditory cortex, dorsal area (AuD), *c-fos* mRNA expression in the ESP1+USVs group was higher than in the ESP1+noise group, although it did not survive multiple comparisons correction [Figure 1B, *t*_(9.96)_ = 2.56, *p* = 0.028]. In other regions, there were no significant differences between the ESP1+USVs and ESP1+noise groups.

#### Prefrontal cortex

**FIGURE 2:**
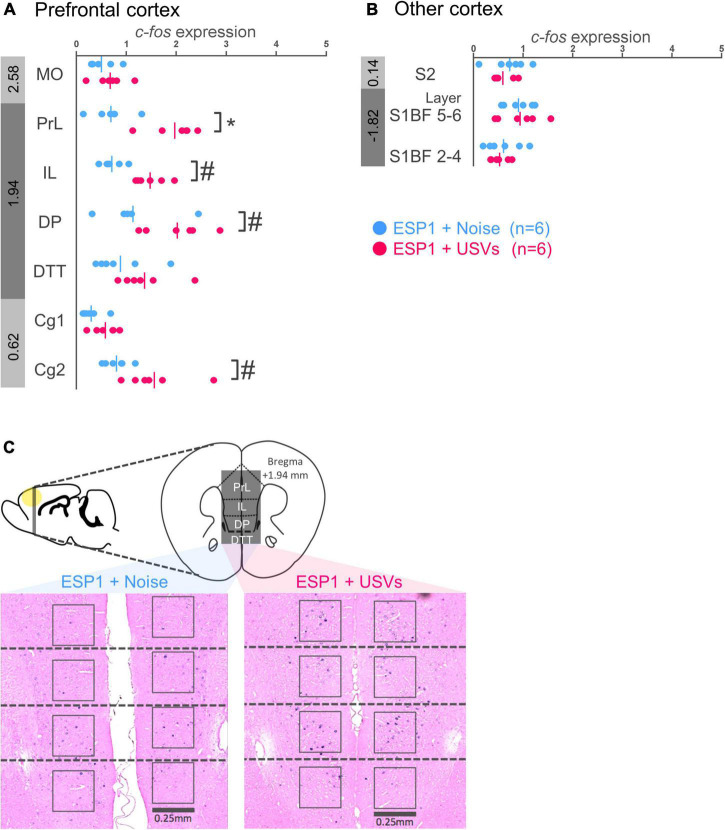
Expression levels of *c-fos* mRNA in the prefrontal cortex **(A)** and other cortices **(B)** of female mice. Blue and red symbols represent individual data for (exocrine gland-secreting peptide 1) ESP1+noise and ESP1+USVs (ultrasonic vocalizations) conditions, respectively. Vertical lines indicate mean values. Numbers in gray bands indicate approximate anteroposterior distance to the bregma (mm). *p < 0.05 (Welch’s *t*-test followed by multiple comparisons correction), ^#^*p* < 0.05 (Welch’s *t*-test; but not significant after multiple comparisons correction). **(C)** Representative images of *c-fos* mRNA expression in the prefrontal cortex. MO, medial orbital cortex; PrL, prelimbic cortex; IL, infralimbic cortex; DP, dorsal peduncular cortex; DTT, dorsal tenia tecta; Cg1, cingulate cortex, area 1; Cg2, cingulate cortex, area 2; S2, secondary somatosensory cortex; S1BF, primary somatosensory cortex barrel field.

In prelimbic cortex (PrL), *c-fos* mRNA expression was significantly higher in the ESP1+USVs group than in the ESP1+noise group [Figures 2A,C, *t*_(9.57)_ = 5.15, *p* = 0.0005]. There was the same tendency in infralimbic cortex (IL), dorsal peduncular cortex (DP), and cingulate cortex area 2 (Cg2) [Figures 2A,C, IL, *t*_(8.90)_ = 5.03, *p* = 0.001; DP, *t*_(9.82)_ = 2.34, *p* = 0.042; Cg2, *t*_(6.41)_ = 2.69, *p* = 0.034]. In the other regions, there were no significant differences between the ESP1+USVs and ESP1+noise groups.

#### Other cortices

There were no significant differences between the ESP1+USVs and ESP1+noise groups in any region (Figure 2B).

#### Lateral septal nucleus and thalamus

**FIGURE 3:**
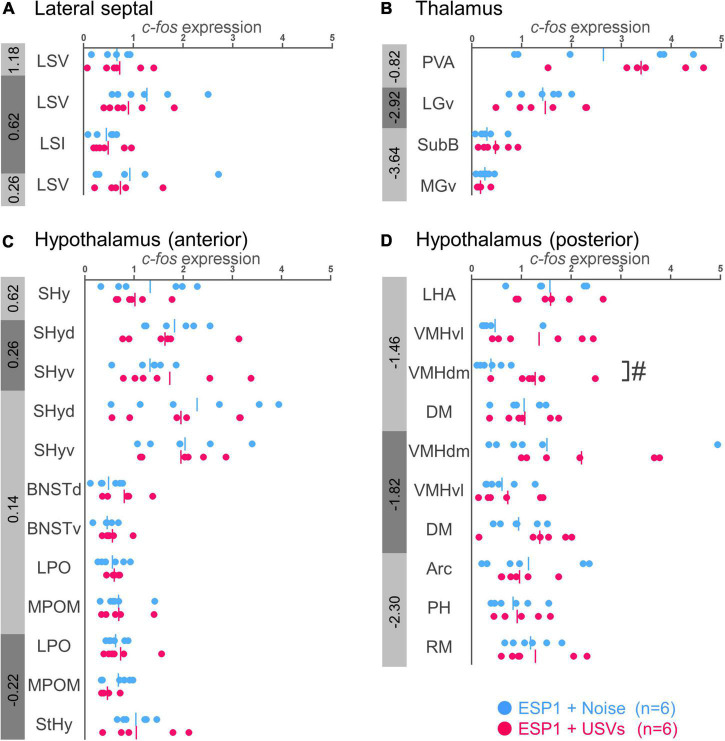
Expression levels of *c-fos* mRNA in the lateral septal nucleus **(A)**, thalamus **(B)**, and hypothalamus **(C,D)** of female mice. Blue and red symbols represent individual data for (exocrine gland-secreting peptide 1) ESP1+noise and ESP1+USVs (ultrasonic vocalizations) conditions, respectively. Vertical lines indicate mean values. Numbers in gray bands indicate approximate anteroposterior distance to the bregma (mm). ^#^*p* < 0.05 (Welch’s *t*-test; but not significant after multiple comparisons correction). LSV, lateral septal nucleus, ventral part; LSI, lateral septal nucleus, intermediate part; PVA, paraventricular thalamic nucleus, anterior part; LGv, lateral geniculate nucleus, ventral part; SubB, subbrachial nucleus; MGv, medial geniculate nucleus, ventral part; SHy, septohypothalamic nucleus; SHyd, septohypothalamic nucleus, dorsal part; SHyv, septohypothalamic nucleus, ventral part; BNSTd, bed nucleus of the stria terminalis, dorsal part; BNSTv, bed nucleus of the stria terminalis, ventral part; LPO, lateral preoptic area; MPOM, medial preoptic nucleus, medial part; StHy, striohypothalamic nucleus; LHA, lateral hypothalamic area; VMHvl, ventromedial hypothalamic nucleus, ventral part; VMHdm, ventromedial hypothalamic nucleus, dorsomedial part; DM, dorsomedial hypothalamic nucleus; Arc, arcuate hypothalamic nucleus; PH, posterior hypothalamic nucleus; RM, retromammillary nucleus.

There were no significant differences between the ESP1+USVs and ESP1+noise groups in any subregion (Figures 3A,B).

#### Hypothalamus

In the dorsomedial part of the ventromedial hypothalamus (VMHdm), *c-fos* mRNA expression in the ESP1+USVs group tended to be higher than in the ESP1+noise group [Figure 3D, *t*_(6.47)_ = 2.96, *p* = 0.023]. In other regions, there were no significant differences between the ESP1+USVs and ESP1+noise groups (Figures 3C,D).

#### Amygdala

**FIGURE 4:**
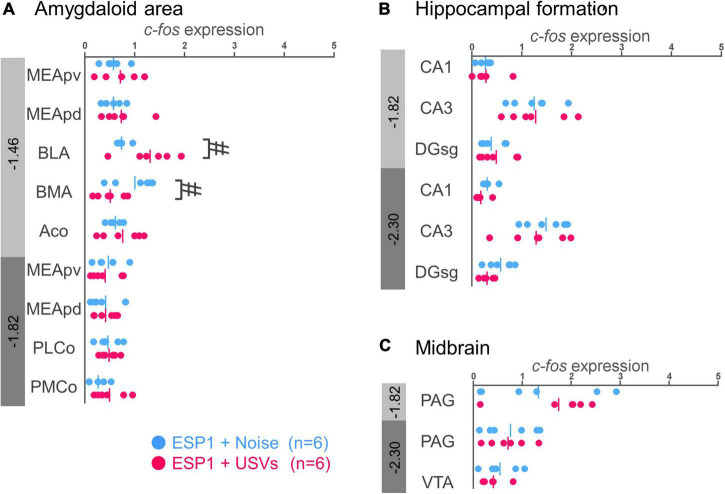
Expression levels of *c-fos* mRNA in the amygdala **(A)**, hippocampus **(B)**, and midbrain **(C)** of female mice. Blue and red symbols represent individual data for (exocrine gland-secreting peptide 1) ESP1+noise and ESP1+USVs (ultrasonic vocalizations) conditions, respectively. Vertical lines indicate mean values. Numbers in gray bands indicate approximate anteroposterior distance to the bregma (mm). ^#^*p* < 0.05 (Welch’s *t*-test; but not significant after multiple comparisons correction). MEApv, medial amygdaloid nucleus, posteroventral part; MEApd, medial amygdaloid nucleus, posterodorsal part; BLA, basolateral amygdaloid nucleus; BMA, basomedial amygdaloid nucleus; Aco, anterior cortical amygdaloid area; PLCo, posterolateral cortical amygdaloid area; PMCo, posteromedial cortical amygdaloid area; CA1, field CA1 of the hippocampus; CA3, field CA3 of the hippocampus; DG-sg, dentate gyrus, granule cell layer; PAG, periaqueductal gray; VTA, ventral tegmental area.

In the basolateral amygdala (BLA), *c-fos* mRNA expression tended to be higher in the ESP1+USVs group than in the ESP1+noise group [Figure 4A, *t*_(5.54)_ = 2.70, *p* = 0.039]. In contrast, in the basomedial amygdala (BMA), *c-fos* mRNA expression tended to be higher in the ESP1+noise group than in the ESP1+USVs group [*t*_(8.84)_ = 2.48, *p* = 0.035]. In other subregions, there were no significant differences between the ESP1+USVs and ESP1+noise groups.

#### Hippocampus and midbrain

No significant differences were found between the ESP1+USVs and ESP1+noise groups in any of the subregions (Figures 4B,C).

#### Correlation between regions

**FIGURE 5:**
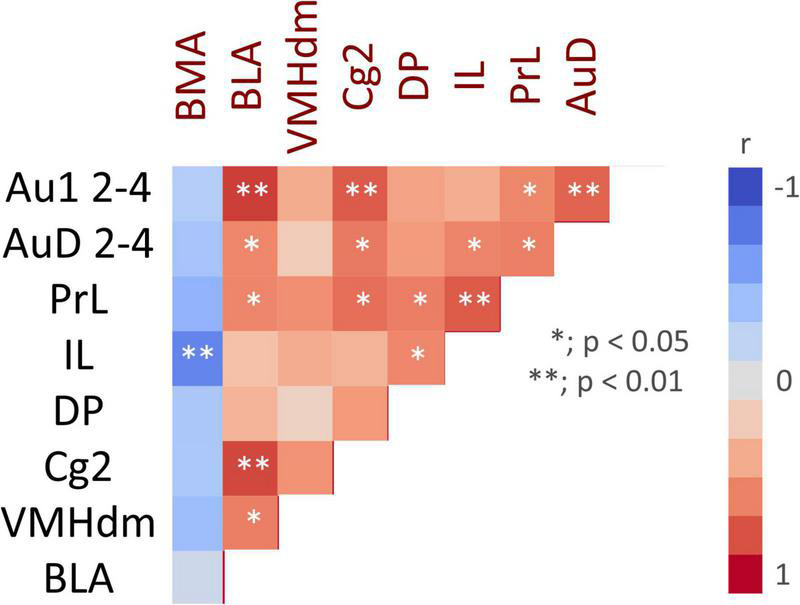
Color map showing pair-wise correlation coefficients for multiple brain regions. Asterisks indicate significant correlation coefficients.

Correlation analysis was performed for regions with significant differences in *c-fos* mRNA expression between the ESP1+USVs and ESP1+noise groups. Although the *c-fos* mRNA expression levels between many of the regions were positively correlated, a negative correlation was obtained in the BMA, with higher *c-fos* mRNA expression in ILs leading to lower *c-fos* mRNA expression in the BMA (Figure 5).

### Experiment 2

Since a previous study reported that medial prefrontal cortex (mPFC) was involved in the development of sound preferences (Yang et al., 2012), mPFC activity was focused in the subsequent analysis. In order to examine effects of olfactory and auditory stimuli on mPFC activities, the 2 × 2 factorial design was used. Female mice were exposed to a combination of pre-exposed ESP1 and USVs (ESP1+USVs group; *N* = 9), pre-exposed ESP1 and noise (ESP1+noise group; *N* = 9), pre-exposed Tris and USVs (Tris + USVs group; *N* = 9), or pre-exposed Tris and noise (Tris+noise group; *N* = 8). Furthermore, the correlation between mPFC activation and female behavior was examined.

#### Analysis of c-fos positive cell counts

**FIGURE 6:**
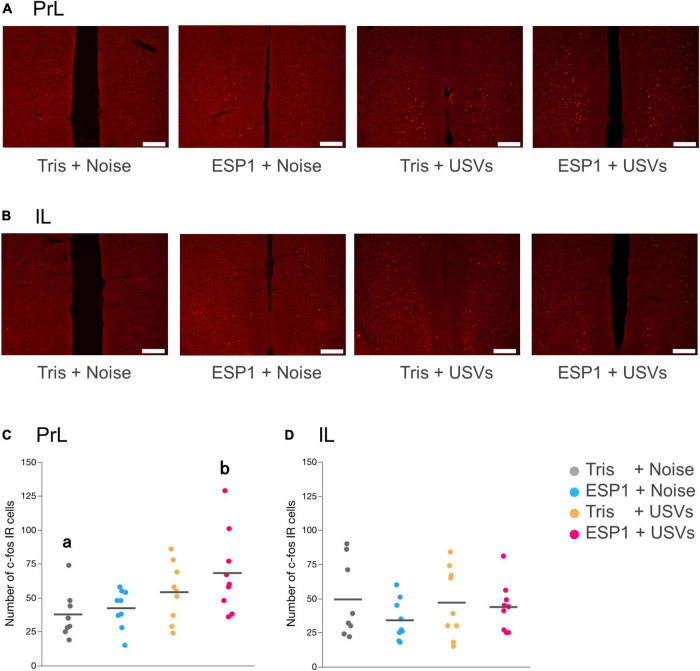
Presentation of ultrasonic vocalizations (USVs) activates PrL neurons. **(A,B)** Representative fluorescent images of c-fos positive cells in PrL and IL. Positive cell is shown in red. Scale bar indicates 0.2 mm. **(C,D)** Number of c-fos positive cells for PrL and IL in the 2 × 2 factorial experimental design. Dots represent individual data. Colors of dots indicate experimental groups. Horizontal lines represent mean values. Different letters indicate significant differences (*p* < 0.05, Tukey HSD test).

Immediate early gene analysis showed that there were c-fos positive cells in mPFC (Figures 6A,B). Two-way ANOVA showed a main effect of USVs presentation (*F*_[1, 31]_ = 8.16, *p* = 0.008), but no interaction of olfactory and auditory stimulus presentation. Multiple comparison tests revealed that the number of c-fos positive cells in the PrL of the ESP1+USVs group was significantly higher than that of the Tris+noise group (*p* = 0.035, Tukey HSD test, Figure 6C). No significant differences were found among the groups in IL (Figure 6D).

#### Behavioral analysis

**FIGURE 7:**
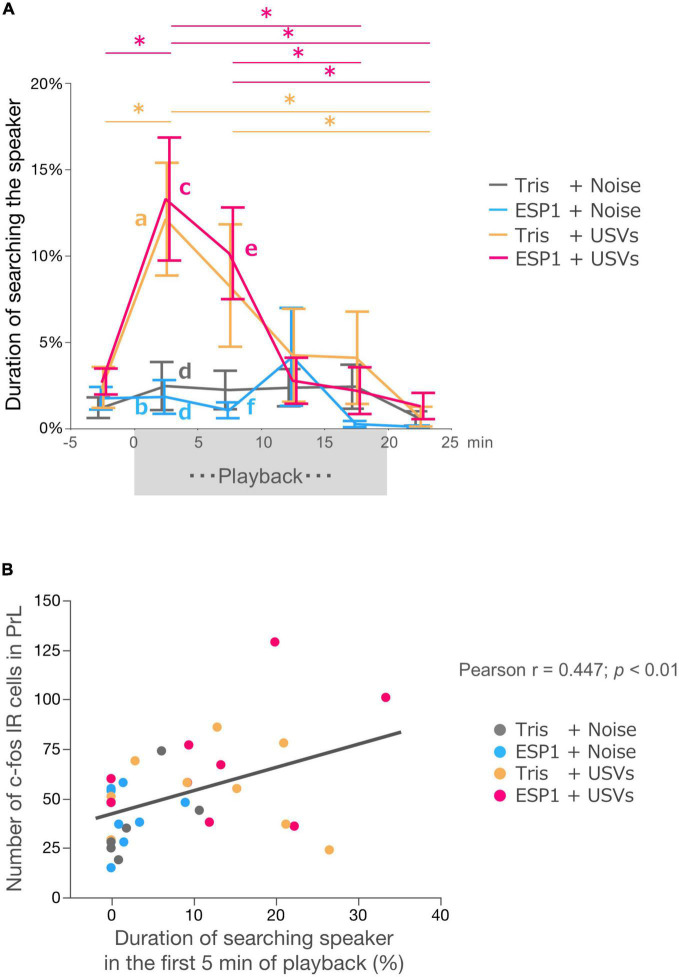
Neural activation in PrL correlates with exploration behavior. **(A)** Duration of speaker exploratory behavior. The whole period was divided into 5-min time windows. Colors represent experimental groups. The (exocrine gland-secreting peptide 1) ESP1+USVs (ultrasonic vocalizations) and Tris+USVs groups showed significantly longer exploration time during the first 5 min and 5–10 min after the start of playback than during the 5 min before playback, 15–20 min after playback, and 5 min after the end of playback. Data points indicate mean ± SEM. *p = 0.05, Two-way ANOVA, Bonferroni test. The Tris+USVs and ESP1+USVs groups had significantly longer speaker exploration times than the Tris+ noise and ESP1+USVs groups during the first 5 min after playback and 5–10 min after playback. Different letters indicate significant differences (*p* < 0.05, Tukey HSD test). **(B)** Correlation between the number of c-fos positive cells in PrL and speaker exploration behavior during the first 5 min of playback (Peason’s *r* = 0.447, *p* < 0.01).

Two-way ANOVA with repeated measures revealed main effects of time windows (*F*_[3.39, 105.12]_ = 8.56, *p* = 0.000017) and stimulus condition (*F*_[3, 31]_ = 4.21, *p* = 0.013). There was also an interaction effect between time windows and stimulus condition (*F*_[10.17, 105.12]_ = 2.41, *p* = 0.012; Figure 7A).

*Post hoc* Bonferroni test showed that in the Tris+USVs group, speaker exploration time during the first 5 min were significantly longer than those during the 5 min before the start of playback (*p* = 0.003) and during the 5 min after the playback (*p* = 0.003). Speaker exploration time between 5 and 10 min after the start of playback was significantly longer than that of 5 min after the end of playback (*p* = 0.039). In the ESP1+USVs group, speaker exploration time during the 5 min after the start of playback was also significantly longer than those during the 5 min before playback (*p* = 0.001), during 15–20 min after playback (*p* = 0.012) and during 5 min after the end of playback (*p* = 0.002). Speaker exploration time between 5 and 10 min after playback was significantly longer than those between 15 and 20 min after playback (*p* = 0.005) and 5 min after the end of playback (*p* = 0.009).

Next, speaker exploration times were compared between the groups within the same time window. Multiple comparison tests showed that during the first 5 min after the playback, the Tris+USVs group significantly increased speaker exploration time compared to the ESP1 + noise group (a vs. b, *p* = 0.039, Tukey HSD test). The ESP1+USVs group also significantly increased speaker exploration time more than the Tris+noise group (c vs. d, *p* = 0.034) and the ESP1+noise group (c vs. d, *p* = 0.018). During the 5–10 min after the playback, the ESP1+USVs group significantly increased speaker exploration time more than the ESP1 + noise group (e vs. f, *p* = 0.044). No significant differences were found among the stimulus condition groups in the other time windows.

#### Correlation analysis of c-fos counts and behavioral analysis

To examine the relationship between neuronal activation in the PrL and female behavior, a correlation analysis was performed. The results showed a positive correlation between the number of c-fos positive cells in PrL and the duration of speaker exploration behavior during the first 5 min of playback (Pearson *r* = 0.4473; *p* = 0.0071, Figure 7B).

### Experiment 3

**FIGURE 8:**
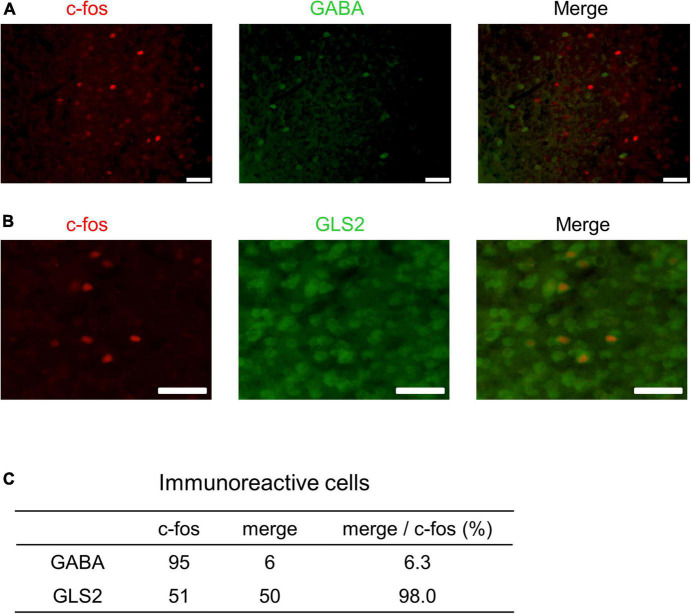
Double immunohistochemistry of c-fos and markers for excitatory or inhibitory neurons in PrL. **(A,B)** Representative fluorescent images. c-fos positive cells are shown in red; GABA- or GLS2-positive cells are shown in green. Scale bars indicate 50 μm. The percentage of double positive cells in the population of c-fos-positive cells is shown in panel **(C)**.

These results indicate that PrL neurons respond to male USVs in the presence of ESP1 and that the number of c-fos positive cells correlates with speaker exploration behavior. To further determine whether these PrL neurons were excitatory or inhibitory, double immunohistochemical analysis with c-fos and markers of excitatory or inhibitory neurons was performed. While GABA was used as a marker for inhibitory neurons, GLS2 was used as a marker for excitatory neurons (Kaneko and Mizuno, 1994). One B6 female was exposed to ESP1, and presented with USVs. The number of c-fos positive cells and the number of cells co-expressing GABA or GLS2 and c-fos were counted (Figures 8A,B). Although the results were preliminary, the number of c-fos positive cells co-expressing GLS2 was greater than that of c-fos positive cells co-expressing GABA (Figure 8C). This suggests that the majority of the PrL neurons activated by male USVs is excitatory.

## Discussion

This study examined how the presentation of male USVs affected brain activities of female mice. In the PrL cortex, c-fos positive cells increased upon USVs presentation in the presence of ESP1. Furthermore, the number of c-fos positive cells in the PrL cortex correlated with approach behaviors for male USVs, such as speaker exploration time. Finally, our preliminary results suggest that the majority of the c-fos positive cells in the PrL cortex is excitatory glutamatergic neurons. Because our sample size was small in Experiment 1, it is possible that we lacked statistical power, resulting in no significant differences after multiple comparisons correction for several brain regions that had *p*-values below 0.05. Thus, we also discuss such brain regions below.

### Medial prefrontal cortex

The PrL was detected as a region significantly activated by the presentation of male USVs. The same tendency was observed in the other mPFC areas such as IL, DP, and Cg2, although these regions did not survive multiple comparisons correction. It has been reported that the sound environment during the critical period affects sound preferences as an adult in mice, and that neural activity in the mPFC is involved (Yang et al., 2012). This suggests that the mPFC plays an important role in the formation and development of sound preferences in mice. In addition, our previous studies have shown that female mice’s preference for male USVs of different strains is acquired during childhood by using cross fostering experiments (Asaba et al., 2014). The present results suggest that mPFC activity may also underlie male USVs preferences of female mice.

There was a positive correlation between the neural activity of PrL neurons and female speaker exploratory behavior. This result indicates that increased neural activity of PrL induces female exploratory behavior to a sound source. Previous studies proposed that the mPFC is thought to be involved in higher cognitive functions such as sensory integration, attention switching, working memory, and decision-making (Gill et al., 2000; Öngür and Price, 2000; Kvitsiani et al., 2013). This suggests that the mPFC may integrate multimodal sensory information and facilitate female exploratory behavior.

Our results suggest that the majority of PrL neurons responsive to the presentation of male USVs are glutamatergic, but not GABAergic. While inhibitory GABAergic neurons are spineless non-pyramidal cells, excitatory glutamatergic neurons are pyramidal cells and transmit information to other areas. The correlation analysis of *c-fos* mRNA showed that there was a positive correlation between PrL, BLA, and VMHdm activities. Although we were unable to determine efferent projections from the PrL, the results of the correlation analysis suggest that PrL, BLA, and VMHdm may form a functional unit that processes multimodal sensory information to exhibit female preference behavior.

### Auditory cortex

A previous study has shown that, by using the optical imaging technique, a part of the primary auditory cortex, the dorsomedial field, is responsive to USVs (Tsukano et al., 2015). In this study, the number of c-fos positive cells in the auditory cortex tended to be higher in female mice exposed to male USVs. However, since the size of the dorsomedial field is relatively small in the coronal sections, it was difficult to tell if the activated neurons in the auditory cortex were within the dorsomedial field. More refined analysis is needed to draw a robust conclusion. Previous studies reported that exposure to pup odor sharpened neuronal responses in layers 2–3 of the primary auditory cortex of lactating mice to pup USVs, suggesting auditory-olfactory interactions in the primary auditory cortex (Cohen et al., 2011; Cohen and Mizrahi, 2015). They have further shown that the modulation of balances between cortical excitation and inhibition underlies plastic auditory processing in mother mice. It remains to be determined whether activated neurons in the auditory cortex is excitatory or inhibitory.

### Hypothalamus

While the number of c-fos positive cells in the VMHdm tended to be higher in the USVs group, the number of c-fos positive cells did not differ between two groups in the ventrolateral part of the ventromedial hypothalamus (VMHvl), which has long been implicated in the regulation of female sociosexual behavior (Pfaff and Sakuma, 1979; Yang et al., 2013; Nomoto and Lima, 2015; Inoue et al., 2019). Recently, our study demonstrated that the inhibition of the VMHdm activity reduced, but not abolished, female lordosis behavior (Ishii et al., 2017), suggesting that the VMHdm also plays an important role in female sexual behavior. That study has also shown that the VMHdm receives afferent inputs from the MeA and controls female-specific social behavior. Furthermore, previous studies showed that ESP1 induced c-fos positive cells in the VMHdm of female mice (Haga et al., 2010; Ishii et al., 2017). Extending our previous findings, the present study showed that the VMHdm tended to be activated by the presentation of male USVs. Given that female mice preferred to copulate with male mice that were able to emit USVs more than with devocalized male mice (Nomoto et al., 2018), this suggests that male USVs may facilitate female sexual behavior at least partially by the enhancement of VMHdm activation.

### Amygdala

This study showed that the BLA tended to be activated by the presentation of male USVs. The BLA is known to receive both olfactory and auditory information (Campeau and Davis, 1995; Maren, 1999). It has also been reported that BLA is activated by co-presentation of pup odor and USVs in mothers and drives maternal behavior (Okabe et al., 2013). There were reciprocal connections between mPFC and BLA and this pathway is reported to control emotional behaviors (Mcdonald et al., 1996; Marek et al., 2013). These findings suggest that, together with the mPFC, the BLA may be part of a brain network that processes multimodal emotional information. In contrast, BMA activity was negatively correlated with activities of other brain regions such as mPFC and BLA. A previous study reported that projections from the ventromedial prefrontal cortex to the BMA suppressed anxiety-related behavior (Adhikari et al., 2015). In the present study, decreased activity in the BMA may reflect the anxious state of female mice exploring the speaker zone.

### Brain mechanisms underlying the modulation of female courtship behavior by male ultrasonic vocalizations

The present results showed that male USVs activated the PrL glutamatergic neurons in the presence of ESP1 in female mice and that the number of activated neurons was positively correlated with the duration of speaker exploratory behavior. This suggests that male USVs facilitate the courtship behavior in female mice exposed to ESP1 before the experiment. There are at least two possibilities for the mechanisms underlying the modulation of female courtship behavior. First, ESP1 may directly enhance neural responses to male USVs. A previous study showed that, in the presence of pup odor, oxytocin modulates excitatory-inhibitory balance in the auditory cortex, enhancing the signal-to-noise ratio of pup USVs (Marlin et al., 2015). However, it may not be the case with ESP1 on female courtship behavior because we did not find significant differences in the activities in the auditory cortex between ESP1+USVs and ESP1+noise groups. In addition, since there was a 30-min interval between the presentations of ESP1 and male USVs in this study, it was not likely that ESP1 directly modulated neural responses to the USVs. The second possibility is that ESP1 may facilitate female courtship behavior in an indirect manner such as modulating female’s motivation. Our previous studies have shown that ESP1 facilitates female lordosis behavior (Haga et al., 2010; Ishii et al., 2017) and that ESP1 is necessary for the USVs preferences of female mice (Asaba et al., 2014; Nomoto et al., 2020). These and the present findings may suggest that, when pre-exposed to ESP1, male USVs activate prelimbic glutamatergic neurons, which mediates the enhancement of female motivation for courtship behavior. In accordance with this hypothesis, previous studies showed that mPFC including prelimbic cortex plays an important role in the regulation of emotion and motivation (Cardinal et al., 2002). Future studies involving real-time monitoring neural activities in the PrL may clarify this issue.

## Data availability statement

The raw data supporting the conclusions of this article will be made available by the authors, without undue reservation.

## Ethics statement

The animal study was reviewed and approved by the Ethical Committee for Vertebrate Experiments of Azabu University.

## Author contributions

AA and TM conducted the experiment. KN wrote the initial draft of manuscript and finalized the manuscript. TO and KT supplied the materials. AA, KN, TM, KK, RK, KT, KM, and TK desinged the experiment and revised the manuscript. All authors contributed to the article and approved the submitted version.
